# Health and Demographic Characteristics of Patients Attending a Newly-Opened Medical Facility in a Remote Amazonian Community: A Descriptive Study

**DOI:** 10.3390/medsci6040106

**Published:** 2018-11-26

**Authors:** Lucy Guile, Devon Graham, Anne Jachmann

**Affiliations:** 1 Department of Anaesthesia, Gloucestershire Hospitals NHS Foundation Trust, Sandford Road, Cheltenham GL53 7AN, UK; 2 Project Amazonas, 701 E Commercial Blvd #200, Ft. Lauderdale, FL 33334, USA; devon@projectamazonas.org; 3 Department of Preventive Medicine and Public Health, University of Valencia, 46100 Burjassot, Valencia, Spain; anjach@alumni.uv.es

**Keywords:** rural health, health-seeking behaviour, health beliefs, Amazon, traditional medicine

## Abstract

Peru is a country with wide regional disparities in health. Remote Amazonian communities have high rates of poverty and poor access to health services. There is a lack of data on morbidity and use of health services in the region. We describe a descriptive, cross-sectional study of the demographic characteristics and presenting complaints of attendees to a newly-opened primary care facility in a remote community. This was supplemented by structured interviews of adult attendees to build a picture of sociocultural determinants of health locally, including engagement with traditional forms of medicine. Our study provides novel insights into an under-studied and under-resourced area. We found a young population with a high prevalence of infectious illnesses, particularly dermatological infections - a previously under-recognised source of morbidity in these communities. Poor literacy rates and widespread use of traditional forms of medicine have important implications for the provision of healthcare in this region.

## 1. Introduction

Despite being classified as an upper-middle-income country, Peru has wide regional disparities in health [1]. The Peruvian Amazon is home to some of the most remote communities on the continent. Compared with the national average, Amazonian communities have worse health indicators [2] and higher rates of poverty [3]. In spite of recent improvements in several social determinants of health, significant inequality persists in wealth distribution and access to basic services, particularly in the Amazon and Andean regions [1]. Within the Amazonian population, indigenous families typically suffer from a higher burden of poverty [4], malnutrition and poor access to health services [5]. Loreto is the largest region in Peru, covering almost 30% of the national territory. Despite its size, it contains just 3.3% of the nation’s population [6], reflecting a low population density outside its main city of Iquitos.

Although estimated mortality rates [7] and morbidity rates for certain conditions such as tuberculosis, malaria and dengue [6,8] are published by the government there is a paucity of accurate data on population health in remote Amazonian communities, reflecting both a lack of investment and the geographical isolation of the region. In a 1999 study, 19% of adult inhabitants of the Yanayacu and Orosa Rivers, Eastern Loreto, reported never having seen a doctor before, and 72% reported being unable to see a doctor when needed [9]. Government data from 2015 indicates that there were just 533 doctors, 796 nurses and 425 ‘obstetras’ (women’s health specialists) in Loreto, serving a population of 1,049,364 [6]. The majority of these healthcare workers are based in urban areas. Most rural healthcare workers in Loreto are recent graduates performing a mandatory year of rural service [2].

A small number of studies published within the last two decades have described health risk factors and behaviours [2,3,9,10], but we found only one small retrospective study that recorded presenting complaints of patients attending a rural Peruvian Amazonian healthcare facility [11]. Our study describes presenting symptoms, working diagnoses and treatments at such a clinic, alongside a survey of the use of traditional forms of medicine and behaviours that influence health. The objective is to contribute to a more detailed picture of the health needs and behaviours of rural communities of the Peruvian Amazon.

The Orosa River, located in Las Amazonas district, Loreto, Peru, is home to over 6000 people, scattered amongst indigenous Yagua and non-indigenous communities. The main source of western medical care was previously a government health clinic several hours’ travel away by motorized canoe [12]. In addition, boat-based clinic visits have been conducted by a non-governmental organization, Project Amazonas, since 1988. In January 2017 the river’s first static medical clinic was opened by Project Amazonas following a formal request for this service from local communities. The clinic is a primary care facility, staffed by Peruvian and (intermittently) foreign healthcare workers, which provides healthcare free of charge to the local population. This study describes the patient profile during the first four weeks of the clinic’s operation, including demographic, social, cultural and medical features. It shows a young population with a high burden of infection, particularly skin infections—a previously under-reported source of morbidity. Literacy levels are substantially below the national average and the use of traditional forms of medicine are widespread amongst both younger and older adults. The latter two observations may have important implications for adherence to prescribed medications and treatment plans.

## 2. Materials and Methods

A descriptive, cross-sectional study was conducted. Basic data were collected for attendees over the first four weeks following the Orosa River Clinic’s opening to create a demographic profile of the patient population and to determine the most common diagnoses, investigations and treatment. The STROBE guideline for cross-sectional studies was adhered to [13]. Demographic and consultation data were collected at the time of consultation via a pro forma completed by one of two researchers, both qualified doctors. The consultations were led by a Peruvian doctor. Details of socio-economic background and use of traditional forms of medicine were collected via structured interview for adult patients who consented to this. These interviews were conducted by one of the two researchers. Consultations and interviews were conducted in Spanish. Eligibility criteria for interview were as follows: aged 18 years or over, capacity to consent to interview, well enough to be interviewed (as judged by the doctor leading the consultation), able to communicate comfortably in Spanish. Consent for interview was sought after the consultation concluded. Consent was in written form unless an interviewee could not sign their name, in which case a thumbprint was used. The interview typically took between ten and twenty minutes to complete.

Sample size was determined by number of clinic attendances. If several people (e.g., a family group) presented together then each person who received a consultation was deemed to have had one attendance. If a person re-presented during the study period, then this was counted as a separate attendance. We aimed to collect data about all clinic attendances and interview all consenting adult attendees over the study period. Interview data was collected using a pro forma and participant information sheet designed by the lead researcher. The study protocol was approved by both Project Amazonas and Brighton and Sussex Medical School Research Governance and Ethics Committee (RGEC Ref No. 16/034/CHE). All elements of the interview, information and consent were explained to participants verbally, with written information also provided. Statistical information is routinely provided by Project Amazonas to the Health Directorate of the Regional Government of Loreto (La Dirección Regional de Salud del Gobierno Regional de Loreto) outlining number of people treated and medical conditions encountered during Project Amazonas’ activities, as part of a pre-existing agreement.

Data were entered using Microsoft Office Excel 2016 for Windows 10 (Version 1805, Microsoft Corporation, Redmond, Washington, United States). Statistical analysis was performed using IBM SPSS Statistics Version 20 and 22 (Armonk, New York, NY, United States).

## 3. Results

**Figure 1:**
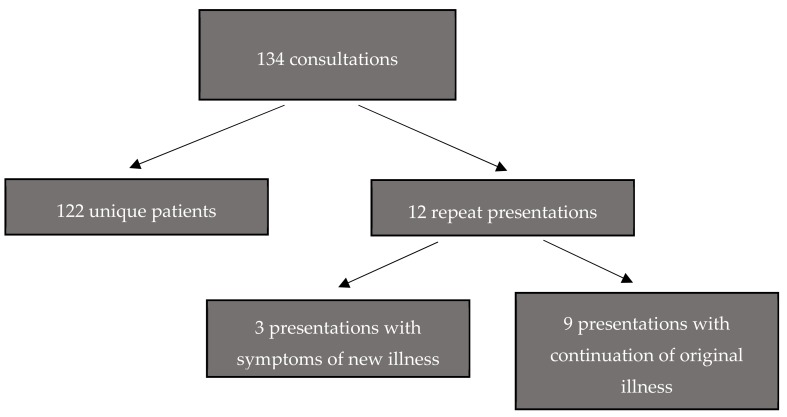
Overview of clinic attendances during the study period.

There were 134 clinic attendances recorded during the study period of 27 January 2017–23 February 2017, representing 122 different patients. The clinic was open on twenty of these 28 days, with availability of the local doctor being the limiting factor. Basic data were collected for all patients on nineteen of the twenty days. 53 of the 61 adult patients who attended were interviewed. Two adult patients declined to participate, two were judged to be too unwell to be interviewed, one needed to transport an unwell relative and one was not asked because her children were restless. No reason was recorded for the remaining two adults who were not interviewed. Nine patients re-presented during the study period with symptoms that were considered to be part of an illness diagnosed at the previous consultation, either spontaneously or as a planned review. Three patients presented with symptoms suggestive of a different illness (Figure 1). Results expressed as percentages show raw numbers in parentheses, for example 50% (61/122). The denominator varies depending on the variable being measured, and in a small number of cases due to missing data.

### 3.1. Demographics

Median age of the 122 patients was 17 years. The oldest attendee was recorded as 76 years, although one elderly woman did not know in which year she had been born, and the youngest was eight months old. An age was not recorded for one other patient. The 2017 Peruvian national census records persons aged under 15 years or over 64 years as dependents [14]. We found that 45.8% (55/120) of clinic attendees were aged under 15 years, and 6.7% (8/120) were aged over 64 years. Additionally, 51.6% (63/122) of clinic patients were female (repeat attendances to the clinic were excluded from sex and age analyses). Of those interviewed, 58.5% (31/53) of adults were co-habiting, 28.3% (15/53) were married, 7.6% (4/53) single, 3.8% (2/53) widowed, 1.9% (1/53) separated. The mean number of people per household was 5.6 (range 2–11).

Ethnic identity was determined by self-identification. Participants were offered a list of options, with the choice of selecting more than one. 18.9% (10/53) of interviewees identified themselves as indigenous, with 13.2% (7/53) identifying as Yagua and 5.7% (3/53) as Cocama. 

Spanish was reported to be the first language of 90.6% (48/53) of the interviewees, with the remaining 9.4% (5/53) reporting this to be Yagua. 13.2% (7/53) were able to speak two languages. One person spoke both English and Spanish, and the other bilingual speakers spoke Yagua and Spanish. All clinic attendees were able to converse comfortably in Spanish.

### 3.2. Symptoms

**Figure 2:**
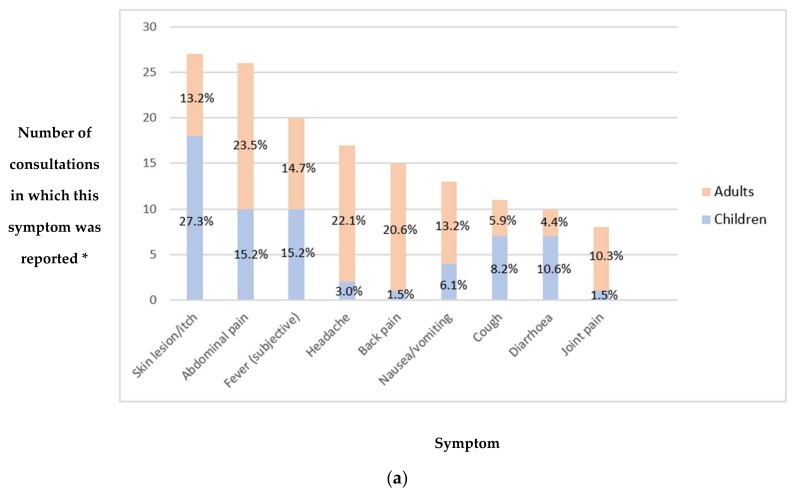

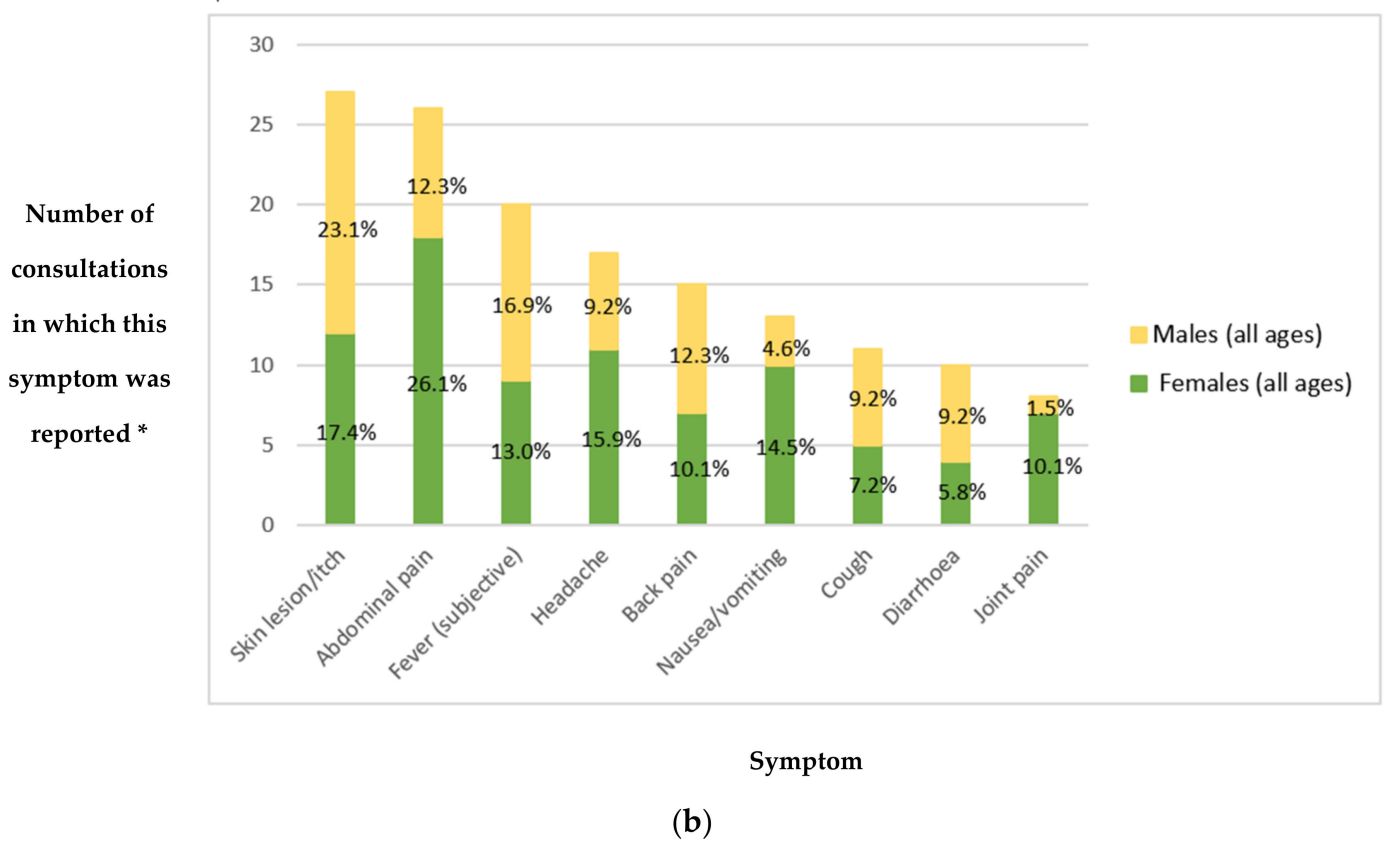
(**a**) Most common symptoms reported by clinic attendees, categorised by age; (**b**) most common symptoms reported by clinic attendees, categorised by sex. * Percentages denote proportion of consultations (categorised by child/adult or female/male) in which this symptom was reported. For example, skin lesion/itch was reported in 27.3% (18/66) of child consultations compared to 13.2% (9/68) of adult consultations, and in 17.4% (12/69) of female consultations compared to 23.1% (15/65) of male consultations.

All reported symptoms (whether offered spontaneously or on specific questioning) were recorded for all patients. Overall, the most common symptom reported was skin lesion or itch, in 20.1% of consultations (27/134). This was more common in children than adults (Figure 2a). This was followed by abdominal pain in 19.4% of consultations (26/134) with a prevalence of 23.5% (16/68) compared to 15.2% (10/66) in adults versus children (Figure 2a), and 26.1% (18/69) versus 12.3% (8/65) in females versus males (Figure 2b). Average number of symptoms reported at first presentation was 1.7 (more than one symptom was reported in 46.3% of consultations (62/134)); this rises to 1.8 if the ten asymptomatic patients who attended to request medication or vitamins are excluded.

### 3.3. Investigations, Diagnoses and Treatment

As is the case in many rural Amazonian health posts, diagnostic tests were very limited. In 83.6% (112/134) of consultations the diagnosis was based on history and/or clinical examination findings alone (excluding bedside observations such as blood pressure and oxygen saturations). No blood tests or imaging were available at the clinic. 14.2% (19/134) of consultations resulted in referral to the nearest government health post, but patients were required to transport themselves there. In the absence of electronic communication systems these referrals took the form of a letter which the patient took with them to the government health post (see Supplementary Materials S1 and S2 for a summary of referrals made).

**Figure 3:**
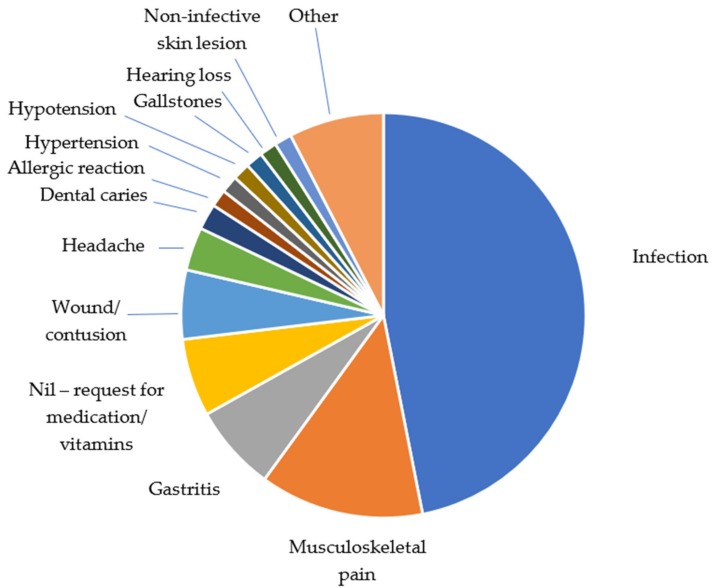
Diagnoses made at the clinic.

The most common diagnosis was infection (46.9% of diagnoses made—68/145), with the most common source the skin (35.3% of infections—24/68 (Table 1a,b), then the gastrointestinal system (26.5%—18/68) (Table 1a), with 61.1% (11/18) of these gastrointestinal infections felt to be parasitic. The next most common diagnoses were musculoskeletal pain (13.1% of diagnoses—19/145) and gastritis (6.9% of diagnoses—10/145) (Figure 3). More than one diagnosis was made in 8.2% of consultations (11/134).

The most commonly administered treatment was oral antimicrobial or antihelminth agents—prescribed in 47.8% of consultations (64/134). Overall, an oral antihelminth agent was prescribed in 29.9% of consultations (40/134), an oral antibacterial in 15.7% (21/134) and an oral antifungal in 4.5% (6/134). A combination of two of these was prescribed in three consultations (2.2%). Five people requested (and received) antiparasitic therapy despite no current symptoms suggestive of infection, and one person was asymptomatic but treated because other household members had evidence of infection, contributing to the high rate of oral antihelminth prescriptions.

Oral analgesia was prescribed in 37.3% of consultations (50/134). Vitamins were prescribed in 27.5% of consultations (37/134), with 18.9% (4/37) of these being prenatal multivitamins for pregnant women, and the others multivitamins administered to both children and adults on the basis of clinical assessment and/or patient request. Topical treatments (most commonly a combined steroid, antifungal and antibiotic cream) were prescribed in 18.7% (25/134), reflecting the high proportion of skin complaints seen.

Other treatments administered were antacids, antihistamines, antitussives and minor procedures such as cleaning and bandaging superficial wounds. Toothbrushes were offered when poor dental hygiene was noted.

### 3.4. Education and Occupation

**Figure 4:**
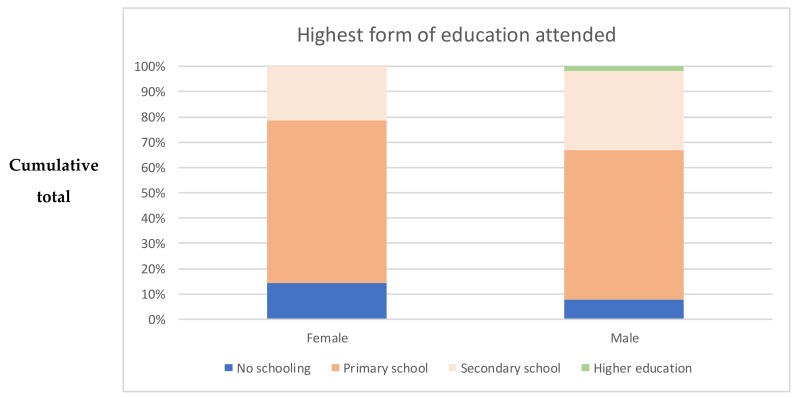
Educational level of females versus males.

Of adults interviewed, 11.3% (6/53) reported having received no schooling at all (14.3% of females (4/28), 8.0% of males (2/25)—see Figure 4). Some had attended primary (62.3% (33/53)) but not secondary school (64.3% of females (18/28), 60.0% of males (15/25)). Some had attended secondary school (26.4% (14/53), 21.4% of females (6/28), 32.0% of males (8/25)), with just one of these (1.9%, male) going on to higher education. Using the Chi-squared test, we found no statistically significant difference in the proportion of males versus females that reported having attended secondary school (*p* = 0.384). Most interviewees reported being able to read well (66.0% (35/53), 60.7% of females (17/28), 72.0% of males (18/25)). A similar proportion—67.9% (36/53)—reported being able to write well (60.7% of females (17/28), 76.0% of males (19/25)). The gender differences in reading and writing ability were not found to be statistically significant.

The majority of interviewees (64.2% (34/53)) reported their primary occupation as agriculture/working in the *chacra*—subsistence farming. This figure represented 57.1% (16/28) of female interviewees and 72.0% (18/25) of male interviewees. A substantial proportion of women (39.3% (11/28)) reported that they cared for their home and family full-time, whilst no men reported this. None of the female interviewees worked in occupations other than subsistence agriculture or caring for their home and family. Other occupations undertaken by men included fishing, shamanism and running a small business. Only one person, a 76-year-old woman, reported that they did not have an occupation. Four other septuagenarian interviewees all worked in subsistence agriculture.

### 3.5. Drinking Water Source and Smoking

Of 53 patients interviewed, 41.5% (22/53) reported that their main source of drinking water was untreated river water, 20.8% (11/53) drank river water but consistently boiled or treated it with chlorine, while 28.3% (15/53) predominantly drank rainwater from a public or private tank, and 9.4% (5/53) bought their drinking water.

The majority of adults (64.2% (34/53)) had never smoked. One person (1.9%) was an ex-smoker. Of the 34.0% (18/53) who currently smoked, 77.8% (14/18) were male. Current smokers mainly reported smoking daily (61.1% (11/18)), with quantities ranging from one to 24 cigarettes per day. Alcohol consumption figures were not accurate, as it became clear during the data collection process that the most commonly-drunk form of alcohol, *masato*, a traditional Amazonian drink, was not reported unless specifically enquired about, as it was considered distinct from imported and other forms of alcohol.

### 3.6. Traditional Medicine

Of adult interviewees, 64.2% (34/53) reported having used traditional forms of medicine, specifically plant-based home remedies — 54.7% (29/53) — and visits to shamans or *curanderos* — 41.5% (22/53). Attitudes varied about the efficacy of traditional versus modern medicine. Several patients reported that certain conditions were only amenable to treatment by shamans. This included the notions of *tunchi*—described by an interviewee as the spirits of bad people who used to live locally and return after death to cause fear and malaise in children—and *cutipa*, which was explained as the transfer of specific negative qualities from an animal to an unborn baby. An example given was that an infant who is ‘*cutipado*’ by a sloth may be slow-moving and lazy. These concepts are described (with variations) in other indigenous and *mestizo* communities of the Peruvian Amazon [15,16]. 

Other interviewees reported typically visiting the government-run clinic in Yanashi when unwell in the first instance, with the shaman as an alternative option if this does not help. Some described plant-based and western remedies as equally effective but took the pragmatic view that it was quicker to take a tablet if readily available than to prepare a remedy, whereas others saw western medicine as superior in general or for specific purposes such as pain relief or treatment of infection, an observation also made by Williamson et al. [3].

## 4. Discussion

The population of clinic attendees was skewed towards a younger age group compared to the general population nationally, with 45.8% of attendees aged under 15 years compared to 26.4% reported by the 2017 national census [14]. It is plausible that children were more susceptible to illness than working-age adults or were more likely to present to the clinic for other reasons, meaning that the age distribution of clinic patients is not necessarily representative of the wider population. Of clinic attendees, 6.7% were aged over 64 years, compared to a national average of 8.4% [14]. Indeed, data from 2016 indicates that Loreto as a whole has a younger population than the national average [6]. Although those aged over 64 years are categorized by the Peruvian national census as dependents, our data indicates that many people continue to work beyond this age, often in small-scale agriculture. It must be noted, however, that 37.7% (20/53) of interviewed adults attended the clinic on foot or by non-motorized canoe (the remainder travelled by motorized canoe) and that this may have been a barrier to attendance for older, frail patients. Home visits to frail patients living close to the clinic could help to mitigate this problem if future staffing of the clinic allows this. These visits would need to be arranged in advance or would require a family member or friend of the patient to attend the clinic in order to request a visit, as no landline telephone network exists locally and mobile phones are not widely used, with coverage very sparse for those who do own a device. Scheduled outreach clinics in more distant communities could also be helpful.

In terms of education, the majority of adults (62.3%) had attended only primary school, with a further 11.3% having received no formal schooling at all. The latter figure is lower than the 19% reported by Nawaz et al. in a 1999 survey that included the same communities [9]. Our finding that 90.6% of interviewees reported Spanish to be their first language was slightly lower than the 94.8% recorded for the district of Las Amazonas as a whole in the 2017 national census [17]. The illiteracy rate in those aged 15 years and over was recorded as 6.0% nationally and 7.7% in Loreto in 2015 [6]. The high proportion of adults in our study who reported being unable to read well (34.0%) undoubtedly has implications for compliance with prescribed medications, as written instructions cannot be relied upon. A prescription was issued in 94.8% (127/134) of study consultations. This finding emphasises the importance of verbally explaining to each patient how to take the medication prescribed for them and of confirming their understanding. It may be appropriate to explore the use of simple compliance aids such as calendar blister packs and pictures to indicate medication timings and course length, both of which have been effective in other settings [18,19].

Our study also indicated that many attendees’ understandings of health and wellbeing are influenced by traditional beliefs and practices, with 64.2% of interviewees having used traditional forms of medicine. This compares with a rate of 71% of attendees to a set of medical boat clinics in Loreto reported by Williamson et al. [3]. Both of these study samples are biased in that they consist of people who have elected to attend a medical clinic, thus it is possible that the proportion of the local population who use traditional medicine is even higher than this. As demonstrated by other studies, traditional health beliefs can influence how people engage with western medical services [20,21,22]. Studies such as that of Mathez-Stiefel et al. [23], who examined the relationship between ‘Western’ and local health knowledge in two rural Andean communities, suggest that these systems of understanding should not be viewed as closed and competing domains, emphasising the ways in which they often complement one another. Other studies have argued that collaboration between these systems promotes health and supports the medical independence of communities [24], with some describing successful public health interventions that focus on the training of traditional healers on the recognition, management and referral of specific medical problems [25]. It is essential that the Orosa River Clinic develops its services with an awareness of the varied understandings within the local population of the determinants and treatment of ill health. A substantial portion of the medical work at the clinic during the study period consisted of providing medical advice and education in a simple, understandable way. Future interventions could involve collaboration with local traditional healers on topics such as the identification of patients who require urgent clinic referral, transmissible diseases and simple hygiene measures such as promoting the boiling of drinking water.

Infection was the most common diagnosis made at the clinic, and skin was the most frequent source. There is a lack of information about morbidity due to dermatological problems in rural Loreto [26]. Collectively, fungal skin conditions were found to be the fourth most prevalent disease globally in 2010, with skin conditions as a whole noted to be the eighteenth leading cause of health burden worldwide, expressed as disability-adjusted life years [27]. It is known that warm, humid climates such as that found in the tropical rainforest are associated with higher prevalence of fungal and bacterial infection [28,29]. A study performed in three urban regional hospitals in the Peruvian Amazon found that the most common dermatological complaint in inpatients was infection, with fungal infection the most prevalent [26], as in our study. As in the overwhelming majority of rural health posts in the Amazon [20], investigations at the study clinic were very limited. The clinic did not have access to microscopy or other basic laboratory facilities. Our study indicates that greater attention to the prevention and treatment of dermatological infections would be of benefit in this region. Provision of a light microscope would aid the diagnosis and management of fungal skin infections, gastrointestinal helminth infection and malaria but would rely on the presence of an appropriately trained staff member.

In keeping with existing literature, gastrointestinal infections were another common cause of morbidity [30]. This is likely to be exacerbated by the finding that 41.5% of interviewed adults reported their main source of drinking water to be untreated river water. Additionally, the consumption of *masato*, a popular beverage which is traditionally prepared by chewing and fermenting cassava, has been linked to the spread of infectious diseases such as Chagas disease [31] and hepatitis B [32].

Identified limitations of the study were that the proportion of chronic problems seen was likely to be higher in the first weeks of the clinic’s opening compared to subsequently—due to the new availability of an accessible local clinic—and that several parameters of interest—such as reading and writing ability—were self-reported rather than objectively verified. We anticipated that participants who were not comfortable speaking Spanish or English (such as monolingual speakers of Yagua or other indigenous languages) could not be interviewed, however, we did not encounter any attendees who were unable to speak fluent Spanish. We also considered the possibility that monolingual speakers of an indigenous language might be dissuaded from attending the clinic due to concerns about ability to communicate with staff. Our literature search unfortunately did not locate any data detailing the proportion of the population of Las Amazonas district that identifies as Yagua or Cocama, which would have helped us to determine whether members of these ethnic groups were more or less likely to access the clinic than members of the non-indigenous community.

An additional limitation of the study was the intermittent conflict between the research role of the interviewers and their position as clinicians. Although consultations were led by a Peruvian doctor who was not involved in the study, if invited to do so, the researchers would offer clinical advice in the interests of the patient. The researchers explained to all interviewees that they were medical doctors. It is plausible that interviewees might have been inhibited from discussing use of traditional forms of medicine in view of this. A future study involving visiting people in their own communities to conduct in-depth interviews on this topic—particularly focusing on the factors that determine whether local remedies or clinic attendance are chosen in the first instance when a particular health concern arises—would be informative. 

Overall, our study demonstrates that infection continues to be a major source of morbidity for communities in the remote Amazon, with dermatological infection an under-recognised problem. Literacy rates are poor compared to the national average, and traditional health beliefs continue to play an important role in local understandings of health and wellbeing. These findings carry important implications for the development of existing and future health initiatives in the remote Peruvian Amazon. Further research on health-seeking behaviour in the context of widespread use of traditional medicine is warranted in order to develop health services that adequately address the population’s needs in a manner that is culturally acceptable and coherent.

## Figures and Tables

**Table:** (**a**)

Source	Number of Diagnoses (% of all Infectious Diagnoses)
Skin/soft tissue	24 (35.3%)
Gastrointestinal	18 (26.5%)
Upper respiratory tract (including ear)	10 (14.7%)
Fever—unclear source	6 (8.8%)
Lower respiratory tract	5 (7.4%)
Genito-urinary	4 (5.9%)
Other	1 (1.5%)

**Table:** (**b**)

Source	Number of Diagnoses (% of all Infectious Diagnoses)
Fungal	13 (54.2%)
Bacterial	6 (25.0%)
Viral (chickenpox)	1 (4.2%)
Mixed bacterial and fungal	1 (4.2%)
Unknown	3 (12.5%)

## Supplementary Materials

The following are available online at http://www.mdpi.com/2076-3271/6/4/106/s1, Table S1: Summary of referrals, Questionnaire S2: Questionnaire-English translation.
