# Supplementary material for: Health and Demographic Characteristics of Patients Attending a Newly-Opened Medical Facility in a Remote Amazonian Community: A Descriptive Study
Source: Med Sci (Basel). 2018 Nov 26;6(4):106. doi: 10.3390/medsci6040106 (PMC6318754; doi:10.3390/medsci6040106)
Supplement: Supplementary file 1 [file medsci-06-00106-s001.zip › Supplementary material 1 - Summary of referrals.docx]

**Summary of referrals**

| **Working diagnosis** | **Number of people** | **Comment** |
| --- | --- | --- |
| Malaria | 3 | Microscopy not available at Orosa River Clinic; No antimalarials stocked as these are provided for free at Centro de Salud de Yanashi (government health post) |
| Tuberculosis | 3 | Imaging and laboratory facilities not available at Orosa River Clinic |
| Biliary colic | 2 |  |
| Pregnancy | 1 | No acute concern but patient had not had routine prenatal checks, which are provided at Centro de Salud de Yanashi |
| Abdominal/pelvic tumour | 1 |  |
| Non-infective skin lesion on face | 1 | Nodular lesion which bled easily; aetiology unclear |
| Wrist injury – suspected fracture | 1 |  |
| Left ankle injury with possible foreign body | 1 |  |
| Disseminated infection or malignancy | 1 | Inguinal and axillary lymphadenopathy; weight loss over previous 12 months |
| Urinary tract infection/Sexually-transmitted infection | 1 | No speculum at clinic to perform gynaecological examination. No urine dipsticks |
| Inflammatory arthritis | 1 |  |
| Vaginal thrush | 1 | Appropriate medication not in stock at Orosa River Clinic |
| Dental caries | 1 |  |
| Chronic hearing impairment | 1 | No DNI (Documento Nacional de Identidad) – National Identity Document. Patient advised to obtain this, then request referral to Hospital Regional de Loreto for assessment and consideration of hearing aids |
