# Supplementary material for: Health and Demographic Characteristics of Patients Attending a Newly-Opened Medical Facility in a Remote Amazonian Community: A Descriptive Study
Source: Med Sci (Basel). 2018 Nov 26;6(4):106. doi: 10.3390/medsci6040106 (PMC6318754; doi:10.3390/medsci6040106)
Supplement: Supplementary file 1 [file medsci-06-00106-s001.zip › Supplementary material 2 - Questionnaire.docx]

**Questionnaire – English translation**

**Demographic data:**

1. Age
2. Date of Birth
3. Sex – male or female
4. Marital status (mark one) -single, married, engaged, separated, divorced, widowed
5. How many children do you have?
6. Where do you live? – name of community
7. Ethnicity – do you consider yourself (mark all that apply): Peruvian, Yagua, Ocaina, Huitoto, Bora, other (please specify which)

**Educational level:**

1. As a child, did you attend school?
2. If yes, to what level did you study? (mark one)

- Attended primary school (but did not complete it)
- Completed primary school
- Attended secondary school (but did not complete it)
- Completed secondary school
- Attended university/technical college

1. Do you speak any language/s in addition to Spanish? Which?
2. What is your first language?
3. Can you read?
4. Can you write?

**Socio-economic background:**

1. What is your occupation?
2. How many people live in your home?
3. How many are aged under 18 years?
4. How is water provided in your home?

- Piped water
- Private water tank
- Public water tank
- Directly from the river (if yes: treated/boiled?)
- Other (please specify what)

1. Do you smoke tobacco? Yes/No/In the past

- How many cigarettes do you smoke per day?
- How long have you smoked for?

1. Do you drink alcohol?

- How often do you drink alcohol?
- What type of alcohol do you drink?

**Access to healthcare:**

1. How long did it take you to get to the clinic today?
2. How did you travel to the clinic?
3. Before the Orosa Medical Clinic opened, what would you do if you needed medical help?
4. Do you take any medications from this or another clinic? If so, what?
5. Do you use any traditional medicines - for example from a shaman or *curandero*, or prepared at home? If so, what?
6. Does modern medicine have any advantages over traditional medicine? If so, what?
7. Does traditional medicine have any advantages over western medicine? If so, what?

************END OF QUESTIONNAIRE************
